# Real-Time Healthcare Data Transmission for Remote Patient Monitoring in Patch-Based Hybrid OCC/BLE Networks †

**DOI:** 10.3390/s19051208

**Published:** 2019-03-09

**Authors:** Moh. Khalid Hasan, Md. Shahjalal, Mostafa Zaman Chowdhury, Yeong Min Jang

**Affiliations:** Department of Electronics Engineering, Kookmin University, Seoul 02707, Korea; khalidrahman45@ieee.org (M.K.H.); mdshahjalal26@ieee.org (M.S.); mzaman@kookmin.ac.kr (M.Z.C.)

**Keywords:** Internet of Things (IoT), eHealth, patch, LED, camera, Bluetooth low energy (BLE), hybrid system, network selection, handover, outage probability

## Abstract

Research on electronic healthcare (eHealth) systems has increased dramatically in recent years. eHealth represents a significant example of the application of the Internet of Things (IoT), characterized by its cost effectiveness, increased reliability, and minimal human effort in nursing assistance. The remote monitoring of patients through a wearable sensing network has outstanding potential in current healthcare systems. Such a network can continuously monitor the vital health conditions (such as heart rate variability, blood pressure, glucose level, and oxygen saturation) of patients with chronic diseases. Low-power radio-frequency (RF) technologies, especially Bluetooth low energy (BLE), play significant roles in modern healthcare. However, most of the RF spectrum is licensed and regulated, and the effect of RF on human health is of major concern. Moreover, the signal-to-noise-plus-interference ratio in high distance can be decreased to a considerable extent, possibly leading to the increase in bit-error rate. Optical camera communication (OCC), which uses a camera to receive data from a light-emitting diode (LED), can be utilized in eHealth to mitigate the limitations of RF. However, OCC also has several limitations, such as high signal-blockage probability. Therefore, in this study, a hybrid OCC/BLE system is proposed to ensure efficient, remote, and real-time transmission of a patient’s electrocardiogram (ECG) signal to a monitor. First, a patch circuit integrating an LED array and BLE transmitter chip is proposed. The patch collects the ECG data according to the health condition of the patient to minimize power consumption. Second, a network selection algorithm is developed for a new network access request generated in the patch circuit. Third, fuzzy logic is employed to select an appropriate camera for data reception. Fourth, a handover mechanism is suggested to ensure efficient network allocation considering the patient’s mobility. Finally, simulations are conducted to demonstrate the performance and reliability of the proposed system.

## 1. Introduction

Electronic healthcare (eHealth) is regarded as one of the most important emerging applications of wireless sensing networks owing to its substantial potential in a wide range of nursing assistance activities. Nowadays, the Internet of Things (IoT) has become a distinguished solution in eHealth [1,2]. Among the promising, advanced IoT applications, eHealth represents a revolutionary segment and offers numerous convenient solutions to patients, doctors, and nursing assistants [3,4,5]. The IoT has a great potential and provides various utilities wherein remote health monitoring of chronic diseases can be regarded as the most significant technology. In this regard, wearable computing technologies are considered to have an incredible influence, because they remarkably eliminate the necessity of continuous physical examination [6]. 

Wearable technologies are extensively developed by researchers to monitor patients with chronic diseases [7,8,9]. These technologies can be demonstrated through wearable armbands or patches embedded in a patient’s body. These patches are designed according to the required operation. A wireless body area network (WBAN) has also been developed, in which healthcare signals are transmitted to the monitoring person. These wearable patches require a biosensor. Different biosensors, such as an electrocardiogram (ECG) [7,10,11], electroencephalogram [12,13], blood pressure [14], and oxygen saturation (SpO_2_) [15] sensors, are developed by researchers. However, the effective wireless transmission of these bio-signals has recently attracted a considerable research interest. The bit-error rate (BER) is highly significant in this regard, because the reliability issue must be addressed with proper attention. In real-time remote monitoring of a patient’s condition (e.g., the heart rate), the data transmission should be almost completely error-free.

Currently, radio-frequency (RF)-based technologies are extensively utilized to accomplish the transmission of healthcare signals to the gateway. A wide range of devices with low-power characteristics, such as Bluetooth low energy (BLE), ZigBee, and IPv6 over low-power wireless personal area networks (6LoWPAN), are considered useful owing to their cost effectiveness and affordability to the majority of people. Among these technologies, BLE is noteworthy for its low power demand and robustness to obstacles [16,17]. After the introduction of BLE 4.0 by a special interest group in 2010, a tremendous research interest has been observed in the area of WBAN, not only for its low-power characteristics, but also for its simple protocol architecture [18]. However, the security of BLE remains questionable [19,20]. For instance, some medical devices are very sensitive to electromagnetic radiation originating from RF-based technologies, which eventually degrade the overall device performance [21]. Moreover, its effect on human health is considerable [22,23]. RF signals are also prone to inter-channel interference and incur a substantial BER. Therefore, to ensure efficient, reliable, and secured healthcare signal transmission, a congruent complementary to RF-based technologies is required.

Optical wireless communication has been actively researched over the last few years. This communication type offers a completely unlicensed spectrum that can be utilized to manage massive future data traffic [24]. Among the communication technologies that use optical spectra, optical camera communication (OCC) is regarded to have significant potential [25]. Herein, a camera image sensor is used to receive an optical signal sent from a modulated light-emitting diode (LED) [26,27,28]. LED flickering is captured in the form of binary data. Moreover, OCC is an excellent solution for both long- and short-distance communications and has several useful features (e.g., high security, excellent signal-to-interference-plus-noise ratio (SINR), and high stability with respect to communication distance variation) [26]. 

OCC is exceptionally secure, as it is almost negligibly affected by the reflected component of the light signal [26]. Unlike RF-based technologies, OCC is less affected by interference. The interfering element can be extracted spatially from the image sensor, because each pixel acts as a photo detector. The BER using OCC is almost zero when the communication distance is short [28,29,30,31]. The achievable data rate of OCC is approximately 55 Mbps, which is also comparable to other low-power-based technologies [25]. Although OCC mainly utilizes the visible light spectrum, it can also operate in the infrared spectrum. Infrared wavelengths are invisible to the human eye and remove the LED flickering that can be bothersome to the patients. In addition, LEDs can be modulated with very low power. By virtue of these characteristics, OCC is a very promising solution in healthcare signal transmission. LED can be embedded in wearable devices, and a camera is utilized to receive the bio-signals. However, data transmission in OCC is instantly terminated if the signal transmission path is blocked by any obstacle. In addition, LED must appear inside the angle-of-view (AOV) of a camera. Furthermore, a direct line-of-sight (LOS) connection is required for successful communication. 

Considering both the utilities and limitations of OCC, we propose a novel hybrid architecture that combines OCC and BLE for real-time healthcare signal transmission to a remote monitor. We consider the transmission of ECG data that particularly focus on heart rate variability. The main contributions of this work are listed below.

- A patch that is connected with an ECG sensor network is proposed. It is constructed using an LED array and a BLE transmitter. The signal is modulated in the LED or BLE and sent to the respective receiver. Static surveillance cameras are utilized to receive the LED data.
- We propose an algorithm that selects the appropriate network in a specific scenario. OCC has been provided with initial priority in the selection mechanism. We employ both single and multiple cameras to compute the selection probability. The AOV can be remarkably increased by using multiple cameras.
- We apply fuzzy logic (FL) to select the most excellent camera. FL is an approach that uses variable truth values ranging from 0 to 1 to generate a certain decision. Different parameters of the OCC performance are investigated while applying FL. The center-of-gravity (COG) method is used to perform defuzzification.
- The selection mechanism is initiated by using a network access request (NAR) generated from the patch circuit. The NAR is produced on the basis of a patient’s current condition. Therefore, power consumption can be minimized when the condition is well controlled.
- To confirm connection reliability, we propose a handover mechanism from OCC to BLE or vice versa. The data are transmitted to a gateway for further transmission to a remote monitor.

The symbols used in our paper are listed in Table 1. The remainder of this paper is organized as follows. Section 2 provides an overview of the current IoT technologies utilized in healthcare. Section 3 introduces the patch circuit and the proposed hybrid infrastructure and presents the channel characteristics of OCC and BLE. Section 4 represents the algorithms for generating a new NAR, network selection for this NAR, and handover from OCC to BLE or vice versa. Section 5 evaluates the performance of the developed selection mechanism in a simulation study. The paper concludes with Section 6.

## 2. Literature Overview

Extensive research has been conducted to determine an efficient data collection and transmission scheme in healthcare over the last two decades. The developed monitoring systems implemented various types of patches for healthcare signal collection. The systems are developed not only for patients with chronic diseases, but also for those who are in critical conditions. Patients’ health conditions that should be monitored in real-time have many types. For example, wearable body sensor networks for blood pressure monitoring were proposed in [32]. A wearable wireless ECG monitoring system was developed in [33], particularly focusing on low power and cost effectiveness. Healthcare systems that monitor diabetic patients were developed in [34,35] by using a smartphone. In the literature [36], a remote monitoring system was proposed to supervise patients developing Alzheimer’s disease by tracking their movement patterns and locations. The same task was implemented in [37] using ZigBee. A wearable monitoring system was also developed to monitor sleep quality by investigating the respiration rate of patients [38]. The types of wearable technologies and monitoring systems for patients with Parkinson’s disease are surveyed in [39].

Patch devices are embedded in patients’ bodies. Thus, it must be ensured that a patient’s natural movements are not troubled by the integration of the device. In addition, the healthcare signals must be transmitted at low power with no or minimal errors. A wide range of low-power devices are used by researchers to transmit healthcare information into another processing unit. Bluetooth was the most widely used owing to its wide availability, robustness to obstacles, and simple protocol structure [16,17]. Bluetooth has now been replaced by BLE, a recent development with low power consumption and a moderate communication range. Meanwhile, additional RF-based technologies are being developed and utilized in remote health monitoring. For example, a survey was conducted focusing on 6LowPAN-based wireless monitoring [40]. Mobility management has had the priority in the literature. A discussion on mobility support using 6LoWPAN is also provided in the literature [41,42]. ZigBee- and ANT-based remote monitoring systems have also been reported [11,33,43]. The collected signals can be processed using a smartphone or personal computer (PC). Table 2 presents a summary of the existing health monitoring systems.

It is worth noting here that the aforementioned systems can suffer from a considerable amount of BER due to interference generated from neighboring devices [31,44]. In addition, the smartphone-based management systems are not very influential in cases where the monitoring person resides in another room or far from the patient. Most studies do not focus on intensive-care scenarios. This type of environment has life or death implications when the connection reliability is questioned. It can be seen from Table 2 that most of the monitoring systems particularly focus on devices with low-power consumption. However, new methods are yet to be proposed to increase reliability. A system focusing on low power, low cost, high security, and enhanced reliability needs to be developed for intensive-care environments.

## 3. System Overview

In our work, we assume an indoor scenario, wherein a patient is confined in an intensive or superficial care unit. An authorized person is remotely monitoring the patient’s health condition. We consider the transmission of ECG data, which particularly focus on heart rate variability. The proposed patch is connected to the ECG data sensing network. The signal is modulated in the LED or BLE and sent to the respective receiver. Static surveillance cameras are utilized to receive the LED data. Eventually, the data are transmitted to the authorized monitoring person using an eHealth gateway.

### 3.1. Patch Connectivity

Our proposed patch circuit is composed of an LED array, LED driving circuitry, and BLE transmitter chip. The patch is embedded in the patient’s arm. Because a direct LOS communication link must be developed between the patch and camera, the patch should be mounted to a completely uncovered part of the body. Our proposed patch circuitry is suitable for monitoring sleeping or unconscious patients. However, the patient may move for some exercise or when he or she goes to a restroom. In these cases, the LED can appear outside the AOV of a camera, which triggers the handover necessity to BLE.

**Figure 1:**
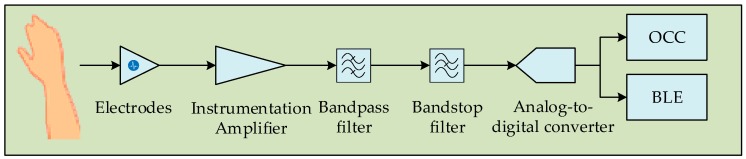
Data acquisition procedure from the patch. OCC: optical camera communication.

The ECG signals can be collected from the body as binary sequences [52]. The considered sensing procedure is similar to those in the literature [7,11]. The ECG signal acquisition is facilitated by using several electrodes. The bio-signals are collected by using a capacitance generated from the electrodes. Then, the signal passes through the instrumentation amplifier. This amplifier reduces the unwanted noise generated in the circuit. In addition, the acquired bio-signal can be naturally weak owing to the regular movement of patients, which is another reason for using this amplifier. In addition, high- and low-pass filters are exploited to pass the signals within a targeted frequency, and a bandstop filter is used to attenuate unwanted frequencies. Thereafter, the signals pass through the analog-to-digital converter (ADC), which presents the digitalized signals. Figure 1 depicts the overall data acquisition procedure.

The ADC is connected with the BLE chip and LED driver circuit. A NAR is generated based on the patient’s condition. The NAR determines whether the bio-signal will be mounted to the BLE chip or the LED driver. At the LED side, the bio-signal is modulated by a small microcontroller circuit. The LED array switching is controlled by a metal-oxide-semiconductor field-effect transistor.

### 3.2. Proposed Hybrid Framework

**Figure 2:**
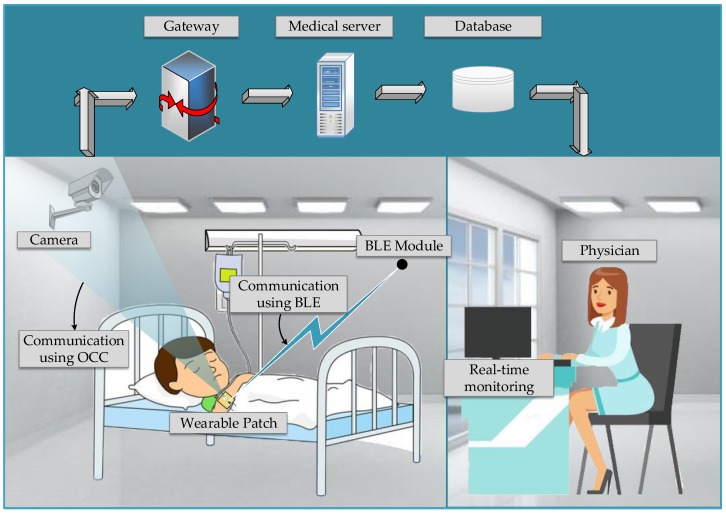
Topology of the proposed health monitoring system.

The proposed hybrid system can be implemented at home, the clinic, the ambulance, or other places. The system operates wherever a patient must be remotely monitored. The number of surveillance cameras in a large-sized room can be high. Multiple patients can be monitored inside the room, thus also confirming the convenience of using multiple cameras. Figure 2 illustrates the monitoring system topology. The camera or BLE receiver collects the data sent from the patch. These collected data are processed to retrieve the original signal, then passed to an eHealth gateway for remote transmission. Several gateway architectures have been proposed [53,54,55]. The data are stored in the eHealth server, which can be accessed from the eHealth database thereafter. Finally, they are transmitted to the remote monitor.

The cameras are assumed to operate by the rolling shutter technique. These rolling-shutter-based cameras are the most popular owing to their worldwide availability and reasonable cost. When a NAR is generated in the patch circuit, the data are transmitted via OCC or BLE (the OCC network is accessed for the initial data transmission). When there is link blockage and high outage probability, the access point for a new NAR switches to BLE. The initial selection or access handover to BLE depends on the position and mobility of the patient.

### 3.3. OCC Channel Model and Data Retrieval Technique

**Figure 3:**
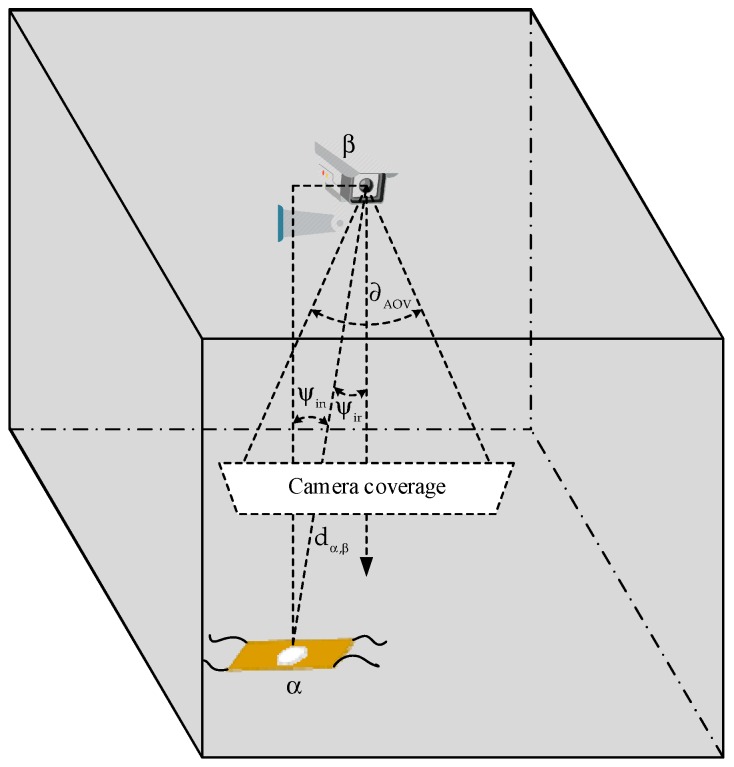
Optical channel model for OCC.

The non-line-of-sight (NLOS) component of the optical signal minimally affects the OCC. The effect is almost negligible when the LED is very small. The data reception can be modeled by the Lambertian radiant intensity [26,56]. The LOS channel model of OCC is depicted in Figure 3. The LED light source and camera are placed in α and β, respectively. The DC LOS channel gain can be represented as follows:

(1) $$G_{\alpha ,\beta }=\{\begin{array}{ll} g_{\mathrm{op}}\cos\left(\psi_{\mathrm{in}}\right)\cos^{m_{l}}\left(\psi_{\mathrm{ir}}\right)\frac{A_{c}\left(m_{l}+1\right)}{2\pi d_{\alpha ,\beta }^{2}} & ,\mathrm{for}\;\psi_{\mathrm{in}}<\partial_{\mathrm{AOV}}\\ 0 & ,\mathrm{for}\;\psi_{\mathrm{in}}\geq \partial_{\mathrm{AOV}} \end{array}$$

where $d_{\alpha ,\beta }$ denotes the Euclidean distance between α and β, $\psi_{\mathrm{ir}}$ indicates the angle of irradiance of the LED, $m_{l}$ signifies the Lambertian emission index (which is a function of the half-intensity radiation angle $\psi_{1/2}$ and formulated as $m_{l}=-\log_{\cos\psi_{1/2}}(2)$), $g_{\mathrm{op}}$ denotes the gain of the optical filter, $\psi_{\mathrm{in}}$ is the angle of incidence, and $A_{c}$ is the area of a projected image on the image sensor. If the pixel edge length is ρ, $A_{c}$ can be expressed as

(2) $$A_{c}=\frac{A_{l}f_{0}^{2}}{\rho^{2}d_{\alpha ,\beta }^{2}}$$

where $A_{l}$ denotes the entire area of the LED that is active to send the optical signals, $f_{o}$ is the focal length of the camera, and $\partial_{\mathrm{AOV}}$ indicates the AOV of the camera.

OCC is less affected by interferences generated from neighboring light sources owing to the nature of the image sensor. The interfering elements can be spatially separated by applying region of interest (ROI) techniques. Thus, OCC offers excellent SINR, which is represented as follows:

(3) $$\eta \left(d_{\alpha ,\beta }\right)=\frac{{\left(\nu P_{t}G_{\alpha ,\beta }\right)}^{2}}{\sum_{i=0}^{N}{\left(\nu P_{t}G_{j,\beta }\right)}^{2}+N_{0}f_{r}}$$

where $P_{t}$ denotes the transmitted optical power, ν is the optical-to-electrical conversion efficiency, $N_{0}$ is the noise spectral density, and $f_{r}$ is the camera sampling rate. In addition, N is the total number of neighboring light sources and $G_{j,\beta }$ is the dc gain of a specific interfering light source.

The LED projected image in the camera is analyzed frame by frame to retrieve the actual data. When the LED is activated, it can be detected by the camera. Computer vision techniques have also been developed recently for object detection [57,58]. We employ a convolutional neural network (CNN) to detect the ROI using the camera. First, the camera captures a series of image frames. Then, a pre-trained CNN is applied to detect the actual ROI to reduce the complexity. Considering that the size of the LED and the transmitted optical signal power are very small, applying CNN is effective and achieves less detection error. The image frames are examined by converting the pixels into grayscale. Subsequently, a certain threshold is set, and the images are binarized, resulting in the appearance of bright pixels that only contain the LED image. Because we use cameras with rolling shutters, the LED image will appear as dark and bright strips because of the “on” and “off” states of the LED. The width of the strips is the function of the LED modulation frequency and the read-out architecture of the camera. By analyzing the width and the number of strips, the data are extracted as binary bits. The total number of strips projected inside the image sensor can be represented as follows [26]:

(4) $$\Gamma =\frac{A_{l}f_{o}^{2}\left(f_{\mathrm{on}}+f_{\mathrm{off}}\right)t_{r}}{\rho^{2}d_{\alpha ,\beta }^{2}}$$

where $f_{o}$ is the focal length of the camera, $t_{r}$ denotes the read-out time of a pixel of the camera, ρ is the edge length of a pixel, and $f_{\mathrm{on}}\;\mathrm{and}\;f_{\mathrm{off}}$ denote the ON and OFF frequencies of the LED, respectively.

The appearance of the full LED to appear inside the image sensor is not necessary for successful communication. Particularly, the strips have a minimum number, denoted by $\Gamma_{\min},$ that should be formed to retrieve the transmitted bits. $\Gamma_{\min}$ is formulated as follows:

(5) $$\Gamma_{\min}=\underset{d_{\alpha ,\beta }}{\arg\min}\left(\Gamma \right)$$

### 3.4. BLE Path Loss Model

The signal power at the BLE receiver is given by [59]

(6) $$P_{\mathrm{rb}}=P_{0}d_{r}{}^{-\upsilon }$$

where $P_{0}$ is the received power at a reference distance from the transmitter, $d_{r}$ is the communication distance, and γ is called the distance power gradient.

The path loss for BLE is expressed as [59]

(7) $$L_{p}=10\log\left(P_{\mathrm{tr}}\right)-10\log\left(P_{0}\right)+10\gamma \log\left(d_{r}\right)$$

where $P_{\mathrm{tr}}$ signifies the transmitted power.

## 4. Proposed Methodologies

### 4.1. FL Employment

As discussed previously, an excellent BER is more important than the data rate in eHealth applications. The network’s capacity is almost negligible, because the monitoring system does not require a similar data rate to voice or video calling. However, the outage probability, which is a function of the achievable SINR, should be given significant attention. The link blockage probability for OCC is also important for reliable communication. Based on these issues, the major performance factors for OCC are regarded as the average SINR, instantaneous received power, and communication distance. These features should be inspected and analyzed before finalizing OCC as the data transmission system. However, setting a certain threshold value of the parameters that should be considered while selecting OCC is particularly inconvenient. Therefore, we envisage FL to assist in the selection mechanism.

FL is a computing approach that utilizes degrees of truth values ranging from 0 to 1 [60,61] rather than using only “true or false” when making decisions. We employed the Mamdani fuzzy inference system to assess the selection process of OCC. Three steps are considered in FL, namely fuzzification of performance parameters, assessment of different “if/then” rules, and defuzzification. Fuzzification is a process that transforms crisp inputs into fuzzy output quantities. The inputs are fuzzified by using numerous fuzzifiers, also referred to as membership functions (MFs) [62]. These functions are utilized to represent a fuzzy set graphically. MFs have different types. We used MFs with different numbers of breakpoints for various inputs. 

**Figure 4:**
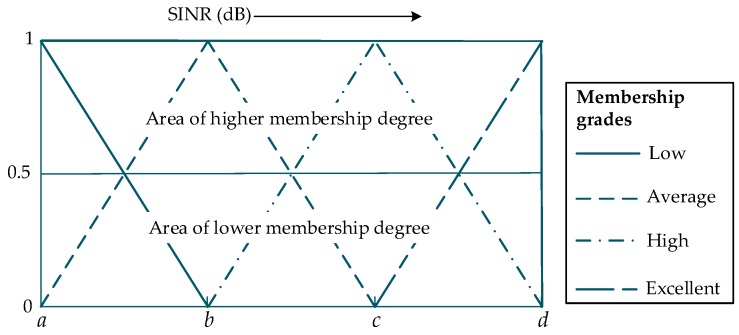
Fuzzification process of SINR with 4 membership grades.

Four input parameters are considered in the fuzzification process. We chose these parameters based on their effects on the OCC performance. The fuzzification process of SINR is illustrated in Figure 4. Four membership grades are chosen, such as low, average, high, and excellent, ranging from −15 to 45 dB. The selected values of the breakpoints *a*, *b*, *c*, and *d* are −15, 0, 15, and 30 dB, respectively. Among the parameters, the fourth parameter that we considered is the number of strips projected inside the image sensor. The significance of this parameter is indisputable when considering OCC for data transmission. As discussed in Section 3, below $\Gamma_{\min}$, the data bits cannot be extracted, although the LED image is projected in the image sensor. We performed several experiments on the training data and eventually chose the MFs. The grade breakpoints of the MFs were selected based on the variations of the parameters with distance prior to outage. Eventually, we selected four triangular MFs for the fuzzification of the SINR and three each for the other input parameters.

The fuzzification process is followed by the assignment of “if/then” rules. The rules are generated on the basis of the considered indoor scenario. The assumption of a patient’s movement was apparent while establishing the rules, because the receiving camera or BLE receiver is a static object. However, mobile robots can be utilized for remote monitoring, which becomes the only case based on which the rules can be employed. We performed the evaluation of the rules by several “anding” operations. The output is denoted by five triangular MFs to obtain a precise result. Finally, the defuzzification step generates a score based on the evaluation rules, which is represented by a crisp value. The inputs can be defuzzified by various methods, such as centroid, bisector, smallest maximum, and largest maximum. We adapted the centroid method owing to its superiority among the other methods. The obtained score, also defined as the selection score (SS), is represented by the following equation.

(8) $$\lambda =\frac{\int u\mu \left(u\right)du}{\int \mu \left(u\right)du},\;\left[\lambda \in R:0\leq \lambda \leq 1\right]$$

where u represents sample input, μ(u) is the MF, and **R** is the universal set of real numbers. 

Based on the values of λ, a particular camera is selected for communication. However, as discussed previously, if the number of strips projected inside the image sensor is below $\Gamma_{\min}$, the communication is instantly terminated. Therefore, scores will not be considered in this circumstance. 

### 4.2. New NAR Generation

A new NAR initiation strictly depends on the patient’s condition. Remotely monitoring a patient is energy inefficient, even when the patient’s health condition is completely normal and has almost no retrogradation possibility. The normal heart rate of individuals in different ages has different ranges [63,64]. Based on the heart rate variability, an instantaneous condition factor is introduced, which will be utilized to initiate a new NAR. Algorithm 1 describes the process of initiating a new NAR. This condition factor is a variable denoted by $\zeta_{c}$ ranging from 0 to 1. It will determine the scheduling process of the new NAR. The normal heart condition will be given a value of $\zeta_{c}$ equal to 1. Two threshold values of $\zeta_{c},$ namely $\zeta_{c\_1}\;\mathrm{and}\;\zeta_{c\_\min},$ which denote the first and second thresholds, respectively, are evaluated. Particularly, these values are naturally variable and strictly depend on the patient’s heart condition. In most remote monitoring cases, a patient is specified with an expected heart rate by the monitoring person. A heart rate close to the optimal extent represents the value of $\zeta_{c}$ close to unity. When the rate decreases or increases from the optimal value, the value of $\zeta_{c}$ decreases. 

**Algorithm 1:** Initialization of the new network access request (NAR).
NARGeneration ()
**Begin**
set time = 0;**if**$\zeta_{c}$ > $\zeta_{c\_1}$ then  put the system in idle mode;**else if**$\zeta_{c\_\min}$ < $\zeta_{c}$ > $\zeta_{c\_1}$ then  Initiate new NAR;  Activate patch;  Set interval = τ seconds
**else**
  Initiate new NAR;  Activate patch;  Set τ=0;
**end if**

**End**



As $\zeta_{c}$ decreases, it eventually reaches its first threshold. As long as it does not reach $\zeta_{c\_1},$ the system will be retained in idle mode, in which the patch is always deactivated. Consequently, the overall power consumption will be reduced. Note that the heart condition of the patient remains completely under control below $\zeta_{c\_1}.$ If the value of $\zeta_{c}$ is lower than $\zeta_{c\_1},$ then the patient will be continuously monitored with a regular interval, τ. The interval depends on the closeness of $\zeta_{c}$ to the second threshold. This interval will be reduced if the closeness is high. 

If $\zeta_{c}$ falls behind the second threshold $\zeta_{c\_\min},$ then the patient will be monitored with no interval. The data will be continuously transmitted as long as $\zeta_{c}$ is higher than $\;\zeta_{c\_\min}.$ The patient is less likely to move when the instantaneous condition factor is very low, so continuous monitoring of the patient is convenient. If the instantaneous and target heart rates are respectively $\sigma_{\mathrm{ins}}$ and $\sigma_{\mathrm{tar}},$ then $\zeta_{c}$ is calculated as follows:

(9) $$\zeta_{c}=\{\begin{array}{c} \frac{\sigma_{\mathrm{ins}}}{\sigma_{\mathrm{tar}}},\sigma_{\mathrm{ins}}<\sigma_{\mathrm{tar}}\\ \left(2-\frac{\sigma_{\mathrm{ins}}}{\sigma_{\mathrm{tar}}}\right),\sigma_{\mathrm{ins}}>\sigma_{\mathrm{tar}}\\ 0,\left[\left(\sigma_{\mathrm{ins}}>2\sigma_{\mathrm{tar}}\right)∥\left(\sigma_{\mathrm{avg}}>2\sigma_{\mathrm{tar}}\right)\right] \end{array}.$$

### 4.3. Network Selection for the New NAR

After generating a new NAR, the patch circuit is activated, and the digitalized signal is modulated. The signal is transmitted by using LED or BLE. The number of surveillance cameras can be more than one but particularly depends on the room size. Algorithm 2 summarizes the network-selection mechanism. The selection probability of OCC depends on the distance between the patient and camera. However, the patient should appear inside the camera AOV. Multiple cameras will increase the OCC coverage. The number of cameras is denoted by ξ.

**Algorithm 2:** Access point selection for new NAR.
Selection ()
**Begin**
**if** new NAR is generated then Collect data from body sensor  Activate patch; **if** ξ = 1 **then**
  Accept Camera (1);  **Call** threshold;**else if***ξ* > 1 **then**  **for** j = 2; j < *ξ*    **if**  *λ*(1) > *λ*(j) **then**      Accept Camera (1);      Set *λ*(j) = *λ*(1);      Set j = j + 1;    **else**
      Accept Camera (j);      Set *λ*(j) = *λ*(1);      Set j = j + 1;    **end if**   **end for**
   **Call** threshold (j); **else**    Accept BLE; **end if**
**else**
Keep system in idle mode;
**end if**

**End**
threshold (x)
**Begin**
**if** Γ > Γ_crit_ **then**
 Accept Camera (x);
**else**
 Accept BLE;
**end if**

**End**



Several factors are investigated before permitting data transmission by OCC. The patch LED will be ready for data transmission only when it is detected by a camera. However, when the number of strips projected in the image sensor is less than $\Gamma_{\min}$, the camera cannot extract data even if the LED is inside the AOV. This problem can occur in two circumstances. First, the Euclidean distance is considerably high between the camera and the patient. Second, the projected image appears in a corner of the image sensor, and the camera confronts the projection of a very small part of the LED. In both cases, Γ can be lower than $\Gamma_{\min}$. Hence, the communication can be instantly terminated in this condition. Therefore, a critical threshold of Γ is significantly required to confirm the reliability of the OCC scheme. The threshold is denoted by $\Gamma_{\mathrm{crit}},$ whose value is only above $\Gamma_{\min}.$ The value of $\Gamma_{\mathrm{crit}}$ varies for the cameras with different characteristics. 

Initially, the system checks the value of ξ. When ξ=1, the camera is selected as the transmission network. However, for the high values of ξ, the SS of each camera is investigated. Then, the camera with the highest SS is selected. When a camera is selected for communication, its Γ is immediately verified whether it is lower than $\Gamma_{\mathrm{crit}}$ or not. If yes, BLE will be selected for communication. 

**Figure 5:**
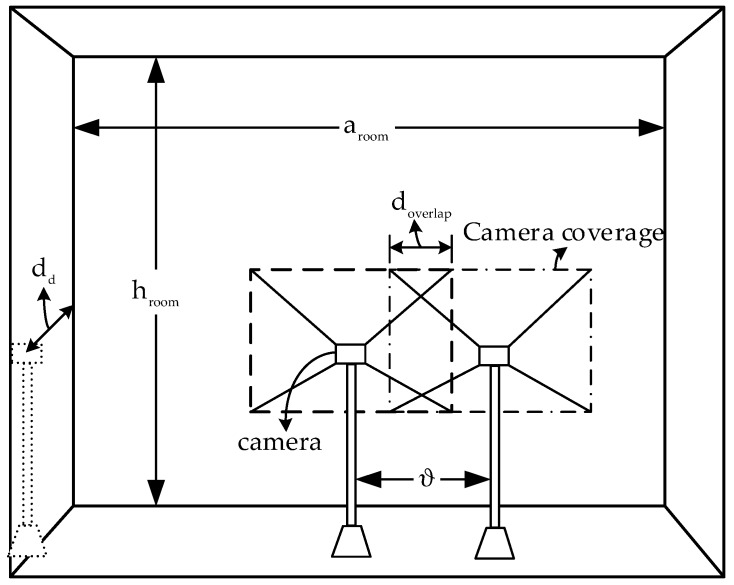
Considered indoor scenario for the selection mechanism.

To theoretically represent the selection probability of each network, we consider a room with a dimension of $a_{\mathrm{room}}\times h_{\mathrm{room}},$ as illustrated in Figure 5. The image sensor dimension of each camera is $p_{\mathrm{im}}\times q_{\mathrm{im}.}$ For simplicity, we assume that each camera is separated with equal distance from each other. The distance is denoted by ϑ. The selection probability of OCC is expressed in the following equation:

(10) $$S_{p\_\mathrm{OCC}}=\frac{\sum_{i=1}^{\xi }\left[\frac{1}{2}p_{\mathrm{im}}q_{\mathrm{im}}{\left(\frac{d_{d}}{f_{o}}\right)}^{2}+d_{d}p_{\mathrm{im}}\vartheta -\psi \right]+\frac{d_{d}p_{\mathrm{im}}}{f_{o}}\left(\frac{q_{\mathrm{im}}d_{d}}{2f_{o}}-\vartheta \right)}{a_{\mathrm{room}}h_{\mathrm{room}}}.$$

Here,

(11) $$\psi =\{\begin{array}{c} \frac{p_{\mathrm{im}}m_{\mathrm{im}}d_{d}}{2f_{o}^{2}},\;\mathrm{for}\;d_{\mathrm{overlap}}\geq \frac{m_{\mathrm{im}}}{2f_{o}}\\ \frac{p_{\mathrm{im}}m_{\mathrm{im}}d_{d}}{f_{o}^{2}},\;\mathrm{for}\;d_{\mathrm{overlap}}<\frac{m_{\mathrm{im}}}{2f_{o}} \end{array}$$

where $d_{d}$ denotes the vertical distance from the LED to the camera and $m_{\mathrm{im}}$ is the minimum part of $q_{\mathrm{im}}$ in which the minimum part of the LED must appear to extract the data bits sent from the LED. 

The minimum LED part depends on the size of the LED and the distance between the LED and image sensor. The minimum area of the LED that should appear inside the image sensor for successful data decoding can be measured using the following equation [26]:

(12) $$A_{m}=\{\begin{array}{ll} 2\int_{r_{l}-r_{m}}^{r_{l}}\sqrt{r_{l}^{2}-x^{2}}dx, & r_{l}>r_{m}\\ \frac{1}{2}A_{l},\; & r_{m}=r_{l}\\ 2\int_{r_{m}-r_{l}}^{r_{m}}\sqrt{r_{l}^{2}-x^{2}}dx, & r_{m}>r_{l}\\ A_{l},\; & r_{m}=2r_{l} \end{array}$$

where $r_{l}$ represents the radius of the LED and $r_{m}$ denotes the minimum portion of the LED that must appear inside the image sensor. 

The quantity $m_{\mathrm{im}}$ ascertains the maximum communication range of the OCC, and $d_{\mathrm{overlap}}$ is the width of the overlapped coverages of two cameras (see Figure 5). The width is formulated as

(13) $$d_{\mathrm{overlap}}=\frac{1}{2f_{o}}\left(q_{\mathrm{im}}d_{d}-\vartheta f_{o}\right).$$

The selection probability of BLE is then simply calculated as

(14) $$S_{p\_\mathrm{BLE}}=1-S_{p\_\mathrm{OCC}}.$$

### 4.4. Network-Switching Policy

As mentioned previously, OCC is a strictly directional LOS technology. If the LOS path of the light signal is blocked, the communication is terminated. Thus, efficient handover between OCC and BLE is required. Network switching is unnecessary in static user scenarios (e.g., patients who are unconscious and/or being transported in ambulances) but necessary when the patient is mobile, because the OCC performance remarkably depends on the LOS Euclidean distance between the LED and camera. The BLE performance is also influenced by user mobility. Efficient handover is thus required for reliable data transmission.

$d_{\alpha ,\beta }$ varies with the patient’s movement. The signal-blockage probability should be considered as it hampers the OCC performance. In addition, when changing the $d_{\alpha ,\beta }$, the SS changes as well, consequently triggering the possibilities for the LED to be projected inside the image sensor with a number of strips below $\Gamma_{\mathrm{crit}}$. The network-switching procedure from OCC to BLE is summarized in Algorithm 3. When $d_{\alpha ,\beta }$ is changed, Γ of the current camera is immediately compared with the threshold. The communication with the current camera is continued if Γ is above $\Gamma_{\mathrm{crit}}$. Otherwise, its λ is compared with the other cameras that simultaneously detect the LED. The camera with the highest λ will be acceptable for communication. However, if no camera can meet the $\Gamma_{\mathrm{crit}}$ requirement, the network will be switched to BLE.

**Algorithm 3:** Network-switching algorithm from OCC to BLE.
O2B_Handover ()Initialization: Communication with camera is in progress;
**Begin**
**if**$d_{\alpha ,\beta }$ is changed **then**  **if** Γ < $\Gamma_{\mathrm{crit}}$ **then**   Initiation of neighboring cameras;   **if** ξ > 1 **then**    **for** j = 1; j < ξ      **if** λ(1) > λ(j) **then**        Accept BLE;        Set λ(j) = λ(1);        Set j = j + 1;      **else if** Γ(j) > $\Gamma_{\mathrm{crit}}$        Accept Camera (j);        Set λ(j) = λ(1);        Set j = j + 1;      **else**        Accept BLE;        Set j = j + 1;      **end if**    **end for**  **else**    Accept BLE;  **end if**   **else**  Continue communication with Camera (1);   **end if**
**else**
  Continue communication with Camera (1);
**end if**

**End**



When the BLE transmitter is activated in the patch circuit, the room cameras are reinitiated at the next change of $d_{\alpha ,\beta }$. If any camera detects the LED, its Γ is immediately compared with $\Gamma_{\mathrm{crit}}$. If the compared Γ is below $\Gamma_{\mathrm{crit}}$, the BLE transmission continues; otherwise, it is replaced by the camera monitoring. If the LED is detected by more than one camera, the λ values of all the in-range cameras are compared, and the network communication switches to the camera with the highest *λ*. Algorithm 4 summarizes the network switching strategy from BLE to OCC.

**Algorithm 4:** Network switching algorithm BLE to OCC.
B2O_Handover ()Initialization: Communication with BLE is in progress;**if**$d_{\alpha ,\beta }$ is changed **then**  Initiation of room cameras;  **for** j = 1, j < ξ   **if** Camera (j) detect LED **then**    **if** Γ(j) > $\Gamma_{\mathrm{crit}}$ **then**      Select Camera (j);      **if** λ(j) > λ(j+1) **then**        Accept Camera (j);        Set j = j + 1;      **else**        Accept Camera (j+1);        Set j = j + 1;      **end if**    **else**      Set j = j + 1;    **end if**  Set j = j + 1;  **else**
    Continue communication using BLE;  **end if**  **end for**

**else**
  Continue communication using BLE;
**end if**

**End**



### 4.5. System Reliability

The reliability of a healthcare data transmission is tremendously significant. Any error in reception can lead to serious issues with patient health. To assess the reliability of the transmission system, several parameters are evaluated in this section, such as outage probability, BER, and quality of service (QoS).

Interference from neighboring RF cells is a main cause of outage in BLE. Current indoor/outdoor infrastructures are installed with abundant RF devices using the 2.4 GHz band, generating a considerable amount of interference. Path loss is another significant contributor to the overall outage probability. BLE outage can be considered to occur below a certain SINR threshold, denoted by χ. The BLE outage probability is calculated as [65]:

(15) $$O_{P\_\mathrm{BLE}}=1-\exp\left[-\frac{\chi }{P_{\mathrm{rb}}}\sum_{k=1}^{N_{b}}I_{T}\left(k\right)\right]$$

where $N_{b}$ denotes the total number of interfering sources with the BLE spectrum, k signifies a specific source, and $I_{T}\left(k\right)$ represents the total power received by the BLE for the specific interfering source.

The interference of neighboring optical sources in OCC is also non-negligible. The interfering elements can be spatially separated from the image pixels, as each pixel acts as a photo detector. However, outage occurs when the LED array is beyond the AOV of the camera. In the scenario of Section 4, the outage probability can be expressed as

(16) $$O_{P\_\mathrm{OCC}}=\frac{\eta \left(d_{\alpha ,\beta }^{\max}\right)\left[a_{\mathrm{room}}h_{\mathrm{room}}-\xi p_{\mathrm{im}}q_{\mathrm{im}}{\left(\frac{d_{d}}{f_{o}}\right)}^{2}+\frac{2d_{d}p_{\mathrm{im}}\left(\xi -1\right)\left(q_{\mathrm{im}}d_{d}-\vartheta f_{o}\right)}{4f_{o}^{2}}+\xi \psi \right]+a_{\mathrm{room}}h_{\mathrm{room}}\eta \left(d_{\alpha ,\beta }^{\max}-d_{\alpha ,\beta }^{\mathrm{crit}}\right)}{a_{\mathrm{room}}h_{\mathrm{room}}\eta \left(d_{\alpha ,\beta }^{\max}\right)}$$

where $d_{\alpha ,\beta }^{\max}$ and $d_{\alpha ,\beta }^{\mathrm{crit}}$ denote the maximum possible distance between the LED and a camera and the maximum communication range when using that camera, respectively. 

The QoS of the healthcare data transmission system significantly depends on how much error-free data is received. Achieving an excellent SINR is indispensable in this regard. Although the data rate is not a highly considerable parameter, a minimum data rate must be ensured in the communication system. We theoretically defined the QoS for the hybrid system as follows:

(17) $$\delta =\{\begin{array}{ll} \frac{\left(2-3\kappa \right)\phi_{\mathrm{ins}}\eta_{\mathrm{ins}}}{2\phi_{\mathrm{tar}}\eta_{\mathrm{tar}}},\; & \left\{\phi_{\mathrm{ins}}<\phi_{\mathrm{tar}}\right\}\&\left\{\eta_{\mathrm{ins}}<\eta_{\mathrm{tar}}\right\}\\ \frac{\left(2-3\kappa \right)\eta_{\mathrm{ins}}}{2\eta_{\mathrm{tar}}}, & \left\{\phi_{\mathrm{ins}}\geq \phi_{\mathrm{tar}}\right\}\&\left\{\eta_{\mathrm{ins}}<\eta_{\mathrm{tar}}\right\}\\ \frac{\left(2-3\kappa \right)\phi_{\mathrm{ins}}}{2\phi_{\mathrm{tar}}}, & \left\{\phi_{\mathrm{ins}}<\phi_{\mathrm{tar}}\right\}\&\left\{\eta_{\mathrm{ins}}\geq \eta_{\mathrm{tar}}\right\} \end{array}$$

where $\phi_{\mathrm{ins}}$ and $\phi_{\mathrm{tar}}$ denote the instantaneous and target data rate, $\eta_{\mathrm{ins}}$ and $\eta_{\mathrm{tar}}$ are the instantaneous and target SINR, and κ represents the BER achieved in reconstructing after reception.

## 5. Performance Evaluation

To simulate our proposed system, we considered a room with dimensions of 5 m×4 m×3 m. Particularly, we employed static surveillance cameras rather than any mobile robot. Table 3 lists the unchanged parameters used in performing the simulations. Note here that any change to luminous parameters will affect the simulation results. All the simulations were executed in MATLAB. 

**Figure 6:**
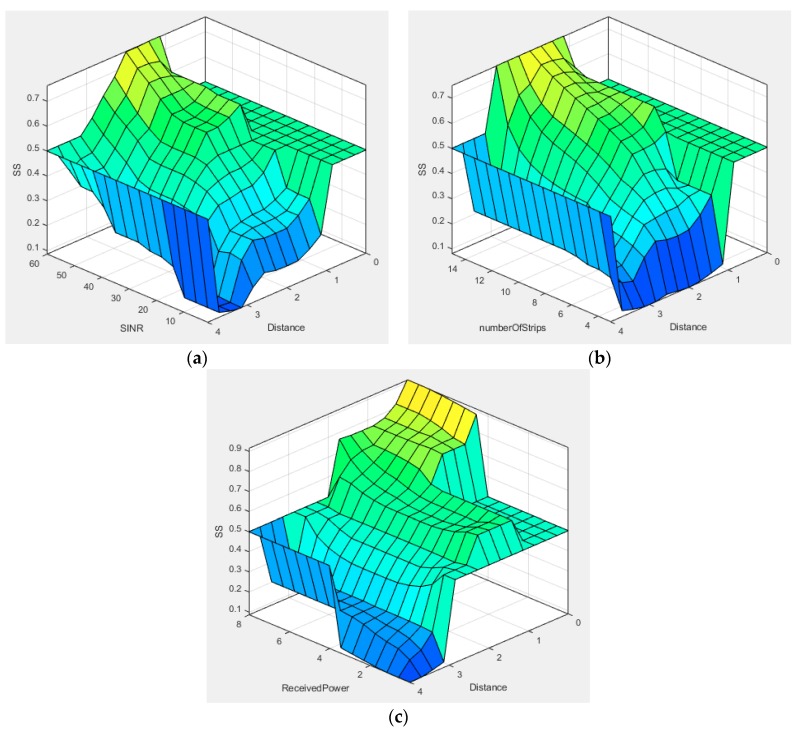
Variation of the selection score (SS) with distance and (**a**) SNIR, (**b**) number of strips, and (**c**) instantaneous received power.

The network selection mechanism for a new NAR particularly depends on the OCC performance. When an LED array is detected by a camera using CNN, λ of the camera is immediately investigated to evaluate the possibility of selecting a camera. Four performance parameters, namely instantaneous received power, number of strips projected in the image sensor, SINR, and LOS communication range, were analyzed to calculate the value of λ. We implemented more than 100 rules to obtain the precise score. The COG method was utilized to calculate λ. Figure 6 depicts the variation of λ in each input parameter with increasing distance using the COG method. To evaluate λ, we considered triangular MFs for all the inputs. The MFs were selected on the basis of a maximum communication range of 4 m.

**Figure 7:**
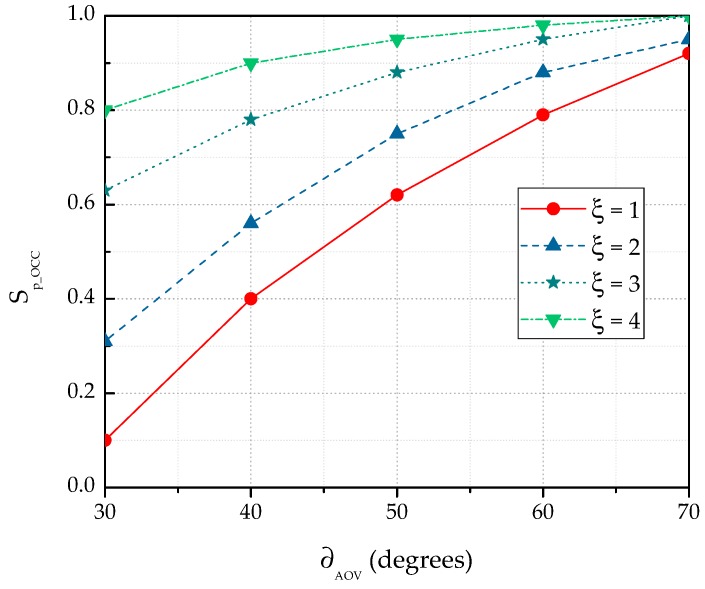
Variation of OCC selection probability for the single- and multiple-camera scenarios.

**Figure 8:**
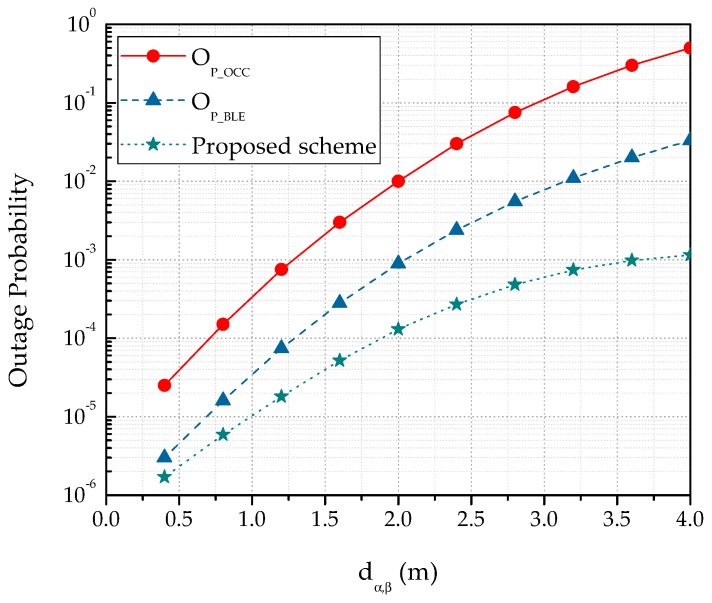
Comparison of the outage probabilities in the OCC, BLE, and proposed schemes.

Noticeably, when increasing the number of cameras, $\partial_{\mathrm{AOV}}$ will be increased to a great extent. Therefore, the probability for the LED to appear inside the camera coverage will also increase. Concurrently, the selection probability for BLE will be reduced. In addition, when coverage of a camera overlaps with another camera, $S_{p\_\mathrm{OCC}}$ depends on the value of $d_{\mathrm{overlap}}.$ Evidently, the cameras can cover a large area when the value of $d_{\mathrm{overlap}}$ is small, hence increasing the selection probability. Figure 7 depicts the variation in $S_{p\_\mathrm{OCC}}$ for a new NAR considering different values of $\partial_{\mathrm{AOV}}$ and ξ. In this simulation, $d_{\mathrm{overlap}}$ and $d_{\alpha ,\beta }$ were fixed at 50 cm and 3 m, respectively. As shown in Figure 7, $S_{p\_\mathrm{OCC}}$ is significantly improved with higher values of $\partial_{\mathrm{AOV}}$. On the other hand, the value of $S_{p\_\mathrm{BLE}}$ is decreased concomitantly. The selection probability also depends on the outage probability of each system. The outage probability for the hybrid system is illustrated in Figure 8. The outage probability of OCC is comparatively high. Several reasons can be addressed for the high outage probability of OCC. First, as most of the current commercial cameras have limited AOV, the overall coverage area is small. Second, data communication requires a LOS connection between the camera and the LED. Third, the optical signal in OCC is almost unaffected by the NLOS component. In Figure 8, we considered both the LOS and NLOS scenarios. However, when monitoring a patient in intensive care, the outage probability of OCC is low because the patient is static and the NLOS caused by the patient’s movement is non-existent. In addition, the outage probability can be minimized by increasing the number of cameras. As is evident in Figure 8, integrating OCC with BLE considerably reduces the outage probability. 

**Figure 9:**
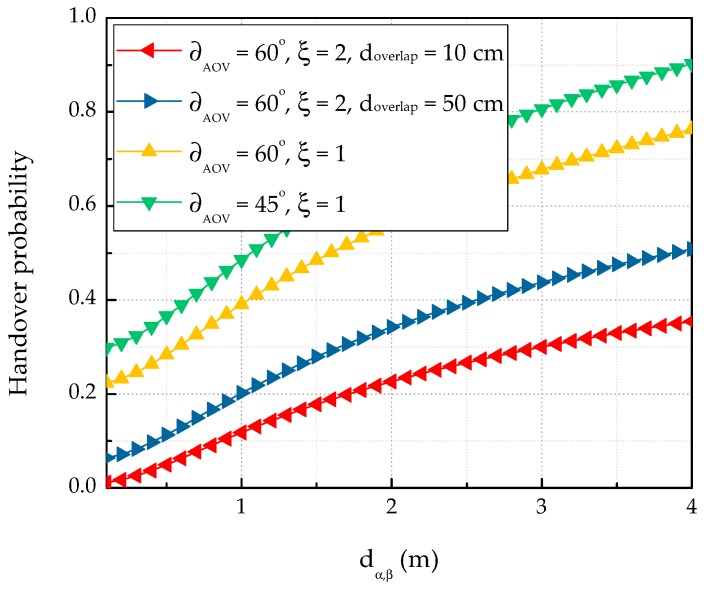
OCC-to-BLE handover probability versus LED-to-camera distance for various $\partial_{\mathrm{AOV}}$ and ξ.

Network switching becomes important when monitoring mobile patients in these scenarios. When patients move, the changing $d_{\alpha ,\beta }$ alters the OCC performance (as discussed above), and hence the possibility of outage in OCC. When an outage occurs, the communication must switch to BLE. Figure 9 plots the OCC-to-BLE handover probabilities in the hybrid system as functions of $d_{\alpha ,\beta }$ for varying $\partial_{\mathrm{AOV}}$ and ξ in the scenario of Section 4. The outage probability significantly decreased with increasing $\partial_{\mathrm{AOV}}$ and ξ and increased with increasing $d_{\mathrm{overlap}}$. Expressed another way, decreasing the $\partial_{\mathrm{AOV}}$ and ξ increases the handover probability from BLE to OCC. 

**Figure 10:**
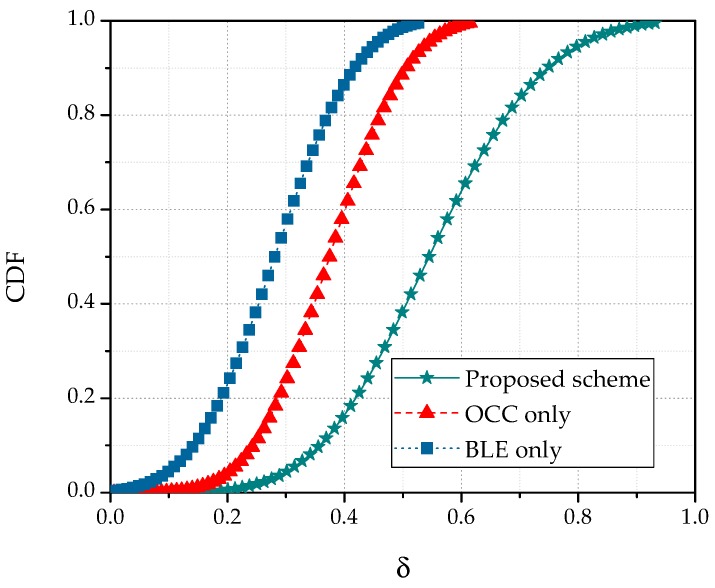
Cumulative distribution function of user quality of service (QoS) in the OCC, BLE, and hybrid schemes.

As discussed earlier, reducing the error in the healthcare information is more important than improving the data transmission rate. Therefore, an excellent SINR is imperative. When the system meets the data rate requirement, its QoS is dominated by the error amount in the received information. In the performance evaluation, we set the target data rate and SINR and estimated the QoS values of the OCC, BLE, and hybrid systems. The cumulative QoS distribution functions in the three systems are compared in Figure 10. The cumulative distribution function (CDF) was calculated over the distance range 0.5–4 m. The CDF of the QoS increased with increasing difference between $\phi_{\mathrm{tar}}$ and $\phi_{\mathrm{ins}}$ and with increasing distance between $\eta_{\mathrm{ins}}$ and $\eta_{\mathrm{tar}}.$ As indicated in Figure 10, the QoS was higher in the proposed scheme than in OCC or BLE. In all the simulations, the spectral density of the OCC noise was assumed to be constant and equal to 10^−21^.

## 6. Conclusions and Future Works

The rapid development of IoT technologies has led to a new dimension in the healthcare field. Remote monitoring of patients’ health conditions using IoT is a promising approach that may result in various convenient solutions to nursing assistants. In this paper, we proposed a wearable ECG monitoring system based on a hybrid OCC/BLE architecture. A patch circuit was suggested where an LED array and BLE transmitter chip are integrated. The patch collects the ECG data from the sensing network and transmits it through a hybrid infrastructure. Depending on the patient’s health condition, a NAR is generated in the patch circuit, and the appropriate network (OCC or BLE) is decided by a network-selection algorithm. The selection mechanism in multiple-camera scenarios is assisted by fuzzy logic. To ensure the safety of both mobile and stationary patients, network-handover mechanisms were proposed. The healthcare information is transmitted to the gateway, where it is stored in an eHealth database before being sent to a remote monitor. The network selection and outage probabilities of each network were mathematically formulated. As confirmed in simulation studies, the hybrid network significantly improved the performance of the data transmission system. However, the performance was evaluated only in an idealized indoor scenario. When multiple patients in a large room are monitored by the same camera, a multiple-input multiple-output setup might be required. In future research, we will test the optimality of the proposed hybrid system over other optical-RF hybrid infrastructures. Other implementation complexities, such as the size and weight of the patch, will also be considered in our future work.

## Figures and Tables

**Table 1:** List of symbols.

Symbols	Definitions	Symbols	Definitions
$G_{\alpha ,\beta }$	Channel gain	γ	Distance power gradient
$g_{\mathrm{op}}$	Gain of the optical filter	$L_{p}$	Path loss using Bluetooth low energy (BLE)
$\psi_{\mathrm{in}}$	Angle of incidence	$P_{\mathrm{tr}}$	Transmitted power using BLE
$\psi_{\mathrm{ir}}$	Angle of irradiance	λ	Selection score
$A_{c}$	Area of the light-emitting diode (LED)-projected image on an image sensor	$\zeta_{c}$	Condition factor
$m_{l}$	Lambertian emission index	$\zeta_{c\_1}\;\mathrm{and}\;\zeta_{c\_\min}$	Thresholds of the condition factor
$d_{\alpha ,\beta }$	Euclidean distance between the camera and LED	$\sigma_{\mathrm{ins}}$	Instantaneous heart rate
$\partial_{\mathrm{AOV}}$	Angle of view of camera	$\sigma_{\mathrm{tar}}$	Target heart rate
$\psi_{1/2}$	Half-intensity radiation angle	τ	Monitoring interval
ρ	Pixel edge length	$\Gamma_{\mathrm{crit}}$	A threshold above $\Gamma_{\min}$
$A_{l}$	Effective area of LED	$p_{\mathrm{im}}\times q_{\mathrm{im}.}$	Image sensor dimension
$f_{o}$	Focal length	ϑ	Distance between two cameras
η	Signal-to-interference-plus-noise ratio	$m_{\mathrm{im}}$	Minimum part of $q_{\mathrm{im}}$ that must appear inside the image sensor in data decoding
ν	Responsivity	$d_{d}$	Vertical distance from LED to camera
$P_{t}$	Transmitted optical power of LED	$d_{\mathrm{overlap}}$	Overlapping distance between two camera coverages
N	Total number of neighboring light sources	χ	Threshold below which BLE outage occurs
$N_{0}$	Noise spectral density	$N_{b}$	Total number of sources interfering with the BLE spectrum
$f_{r}$	Frame rate	$I_{T}$	Power received from an interfering source
Γ	Total number of strips	$d_{\alpha ,\beta }^{\max}$	Maximum possible distance between LED and camera
$f_{\mathrm{on}}$	ON frequency of LED	$d_{\alpha ,\beta }^{\mathrm{crit}}$	Maximum communication range
$f_{\mathrm{off}}$	OFF frequency of LED	$\phi_{\mathrm{ins}}$	Instantaneous data rate
$t_{r}$	Read-out time of a pixel	$\phi_{\mathrm{tar}}$	Target data rate
$\Gamma_{\min}$	Minimum number of generated strips needed for data decoding	$\eta_{\mathrm{ins}}$	Instantaneous signal-to-interference-plus-noise ratio (SINR)
$P_{\mathrm{rb}}$	Power received by BLE receiver	$\eta_{\mathrm{tar}}$	Target SINR
$P_{0}$	Received power from a reference distance by BLE receiver	κ	Bit error rate
$d_{r}$	Communication distance between BLE transmitter and receiver		

**Table 2:** Summary of health monitoring systems developed based on the literature.

Literature	Data Transmission Technology	Monitoring Health Condition	Data Collection and Processing System	Aim of the Work
[7]	BLE	Electrocardiogram (ECG)	Smartphone	Development of a reliable, robust, and low-power system
[11]	ANT	ECG	Personal computer (PC)-based management	Designing a low-power, small-sized, and effective monitoring system
[33]	ZigBee	ECG	PC-based management	Designing a system with long battery life and high-quality signal reception
[35]	IPv6 over low-power wireless personal area networks (6LoWPAN)	Glucose level	PC-based management	Utilizing Mobile-Internet of Things (m-IoT)for diabetes management
[36]	Bluetooth	Detection of Alzheimer’s disease	Bluetooth-enabled monitoring device	Detecting early Alzheimer’s and augmenting life expectancy
[38]	BLE	Sleep	Smartphone	Developing a reliable magnetometer sensor with low power
[43]	ZigBee	Blood pressure	PC-based management	Easy and clear examination of results
[45]	Bluetooth	Blood pressure	Android smartphone	Accuracy enhancement over the existing technologies
				
[46]	3G/WiFi enabled 6LoWPAN	ECG	PC-based management with 6LoWPAN enable edge router	Providing a flexible technological solution for real-time remote monitoring
[47]	Bluetooth and Global System for Mobile Communications (GSM)	ECG	Mobile phone	Continuous monitoring and data acquisition from anywhere
[48]	Bluetooth	ECG	Android smartphone	Developing a non-contact electrode circuit with low power consumption and good signal quality
[49]	2.4 GHz radio and a proprietary protocol	Electromyography (EMG) and oxygen saturation (SpO_2_)	PC or smartphone	Development of a low-power sticking patch with reusable battery and adhesive ingredients
[50]	Bluetooth	Detection of toxic volatile organic compounds	Cell phone	Designing a system with a novel tuning fork sensor with high sensitivity and selectivity
[51]	Bluetooth	Oxygen concentration in breath	Android smartphone or tablet	Design and characterization of a fully wearable system applicable everywhere

**Table 3:** Unchanged system parameters for the simulation.

**OCC parameters**	
Effective LED area, $A_{l}$	7 cm^2^
Half-intensity radiation angle, $\Psi_{1/2}$	60°
Transmit power, $P_{t}$	15 dbm
Gain of optical filter, $g_{\mathrm{op}}$	1.0
Image sensor aspect ratio	3:2 aspect ratio
Pixel edge length, ρ	1.5 µm
Frame rate, $f_{r}$	30 fps
Focal length, $f_{o}$	36 mm (effective)
Responsivity, ν	0.51
**BLE parameters**	
Frequency band	2.4 GHz
Modulation index	0.5
Channel bandwidth	2 MHz
Transmit power, $P_{\mathrm{tr}}$	20 dBm
**Indoor scenario**	
Room dimension	5 m×4 m×3 m
Camera height from ground	1.5 m

## References

[1] Islam S.M.R., Kwak D., Kabir M.D.H., Hossain M., Hossain M., Kwak K. (2015). The internet of things for health care: A comprehensive survey. IEEE Access.

[2] Baker S.B., Xiang W., Atkinson I. (2017). Internet of things for smart healthcare: Technologies, challenges, and opportunities. IEEE Access.

[3] Sawand A., Djahel S., Zhang Z., Naït-Abdesselam F. (2015). Toward energy-efficient and trustworthy eHealth monitoring system. China Commun..

[4] Gope P., Hwang T. (2016). BSN-care: A secure IoT-based modern healthcare system using body sensor network. IEEE Sens. J..

[5] Fan Y.J., Yin Y.H., Xu L.D., Zeng Y., Wu F. (2014). IoT-based smart rehabilitation system. IEEE Trans. Ind. Inform..

[6] Alam M.M., Malik H., Khan M.I., Pardy T., Kuusik A. (2018). A survey on the roles of communication technologies in IoT-based personalized healthcare applications. IEEE Access.

[7] Rachim V.P., Chung W. (2016). Wearable Noncontact Armband for Mobile ECG Monitoring System. IEEE Trans. Biomed. Circuits Syst..

[8] Yama Y., Ueno A., Uchikawa Y. Development of a wireless capacitive sensor for ambulatory ECG monitoring over clothes. Proceedings of the International Conference IEEE EMBS.

[9] Gao Y., Li H., Luo Y. (2015). An empirical study of wearable technology acceptance in healthcare. Ind. Manag. Data Syst..

[10] Majumder S., Chen L., Marinov O., Chen Ch., Monday T., Deen M.J. (2018). Noncontact Wearable Wireless ECG Systems for Long-Term Monitoring. IEEE Rev. Biomed. Eng..

[11] Nemati E., Deen M.J., Mondal T. (2012). A wireless wearable ECG sensor for long-term applications. IEEE Commun. Mag..

[12] Li G., Lee B., Chung W. (2015). Smartwatch-based wearable EEG system for driver drowsiness detection. IEEE Sens. J..

[13] Nakamura T., Goverdovsky V., Morrell M.J., Mandic D.P. (2017). Automatic sleep monitoring using ear-EEG. IEEE J. Transl. Eng. Health Med..

[14] Zheng Y., Yan B.P., Zhang Y., Poon C.C.Y. (2014). An armband wearable device for overnight and cuff-less blood pressure measurement. IEEE Trans. Biomed. Eng..

[15] Yang D., Zhu J., Zhu P. SpO2 and heart rate measurement with wearable watch based on PPG. Proceedings of the IET International Conference on Biomedical Image and Signal Processing.

[16] Tabish R., Mnaouer A.B., Touati F., Ghaleb A.M. A comparative analysis of BLE and 6LoWPAN for U-HealthCare applications. Proceedings of the 7th IEEE GCC Conference and Exhibition (GCC).

[17] Siekkinen M., Hiienkari M., Nurminen J.K., Nieminen J. How low energy is bluetooth low energy? comparative measurements with ZigBee/802.15.4. Proceedings of the IEEE Wireless Communications and Networking Conference Workshops.

[18] Gomez C., Oller J., Paradells J. (2012). Overview and evaluation of bluetooth low energy: An emerging low-power wireless technology. Sensors.

[19] Ryan M. Bluetooth: With low energy comes low security. Proceedings of the USENIX Conference Offensive Technologies.

[20] Das A.K., Pathak P.H., Chuah C.N., Mohapatra d.P. Uncovering privacy leakage in BLE network traffic of wearable fitness trackers. Proceedings of the International Workshop on Mobile Computing Systems and Applications.

[21] Furuhata H. Electromagnetic interferences of electric medical equipment from hand-held radiocommunication equipment. Proceedings of the International Symposium on Electromagnetic Compatibility.

[22] Radiofrequency (RF) Radiation Health Physics Society. https://hps.org/hpspublications/articles/rfradiation.html.

[23] Non-Ionizing Electromagnetic Radiation in the Radiofrequency Spectrum and Its Effects on Human Health. http://www.wireless-health.org.br/downloads/LatinAmericanScienceReviewReport.pdf.

[24] Chowdhury M.Z., Hossan M.T., Hasan M.K., Jang Y.M. (2018). Integrated RF/Optical wireless networks for improving QoS in indoor and transportation applications. Wirel. Pers. Commun..

[25] Goto Y., Takai I., Yamazato T., Okada H. (2016). A new automotive VLC system using optical communication image sensor. IEEE Photonics J..

[26] Hasan M.K., Chowdhury Z., Shahjalal M., Nguyen V., Jang Y. (2018). Performance analysis and improvement of optical camera communication. Appl. Sci..

[27] Ghassemlooy Z., Luo P., Zvanovec S. (2016). Optical camera communications. Optical Wireless Communications.

[28] Shahjalal M., Hossan M.T., Hasan M.K., Chowdhury M.Z., Le N.T., Jang Y.M. (2018). An implementation approach and performance analysis of image sensor based multilateral indoor localization and navigation system. Wirel. Commun. Mob. Comput..

[29] Chen S., Chow C. (2014). Color-shift keying and code-division multiple-access transmission for RGB-LED visible light communications using mobile phone camera. IEEE Photonics J..

[30] Luo P., Zhang M., Ghassemlooy Z., Le Minh H., Tsai H., Tang X., Png L., Han D. (2015). Experimental demonstration of RGB LED-based optical camera communications. IEEE Photonics J..

[31] Hasan M.K., Shahjalal M., Chowdhury M.Z., Jang Y.M. Access Point Selection in Hybrid OCC/RF eHealth Architecture for Real-Time Remote Patient Monitoring. Proceedings of the International Conference on Information and Communication Technology Convergence.

[32] Lin H., Xu W., Guan N., Ji D., Wei Y. (2015). Noninvasive and continuous blood pressure monitoring using wearable body sensor networks. IEEE Intell. Syst..

[33] Spanò E., Pascoli S.D., Iannaccone G. (2016). Low-power wearable ECG monitoring system for multiple-patient remote monitoring. IEEE Sens. J..

[34] Chang S.H., Chiang R.D., Wu S.J., Chang W.T. (2016). A contextaware, interactive M-health system for diabetics. IT Prof..

[35] Bui N., Bressan N., Zorzi M. Interconnection of body area networks to a communications infrastructure: An architectural study. Proceedings of the European Wireless Conference.

[36] Cheng H.T., Zhuang W. (2010). Bluetooth-enabled in-home patient monitoring system: Early detection of Alzheimer’s disease. IEEE Wirel. Commun..

[37] Taub D.M., Lupton E., Hinman R., Leeb S., Zeisel J., Blackler S. (2011). The escort system: A safety monitor for people living with Alzheimer’s disease. IEEE Pervasive Comput..

[38] Milici S., Lázaro A., Villarino R., Girbau D. (2018). Wireless wearable magnetometer-based sensor for sleep quality monitoring. IEEE Sens. J..

[39] Pasluosta C.F., Gassner H., Winkler J. (2015). An emerging era in the management of Parkinson’s disease: Wearable technologies and the Internet of Things. IEEE J. Biomed. Health Inform..

[40] Shahamabadi M.S., Ali B.B.M., Varahram P., Jara A.J. A network mobility solution based on 6LoWPAN hospital wireless sensor network (NEMO-HWSN). Proceedings of the International Conferences Innovation Mobile Internet Services Ubiquitous Computer.

[41] Jara A.J., Zamora M.A., Skarmeta A.F.G. (2010). An Initial Approach to Support Mobility in Hospital Wireless Sensor Networks based on 6LoWPAN (HWSN6). J. Wirel. Mobile Netw. Ubiquitous Comput. Dependable Appl..

[42] Wang X., Zhong S., Zhou R. (2012). A mobility support scheme for 6LoWPAN. Comput. Commun..

[43] Li W.J., Luo Y.L., Chang Y.S., Lin Y.H. A wireless blood pressure monitoring system for personal health management. Proceedings of the IEEE International Conference of the IEEE Engineering in Medicine and Biology Society.

[44] Golmie N., Cypher D., Rebala O. (2005). Performance analysis of low rate wireless technologies for medical applications. Comput. Commun..

[45] Singh M., Jain N. (2016). Performance and evaluation of smartphone based wireless blood pressure monitoring system using Bluetooth. IEEE Sens. J..

[46] Tabish R., Ghaleb A.M., Hussein R., Touati F., Mnaouer A.B., Khriji L., Fadl M. A 3G/WiFi-enabled 6LoWPAN-based U-healthcare system for ubiquitous real-time monitoring and data logging. Proceedings of the 2nd Middle East Conference on Biomedical Engineering.

[47] Tseng K.C., Lin B., Liao L., Wang Y., Wang Y. (2014). Development of a wearable mobile electrocardiogram monitoring system by using novel dry foam electrodes. IEEE Syst. J..

[48] Lin B.S., Chou W., Wang H., Huang Y., Pan J. (2013). Development of novel non-contact electrodes for mobile electrocardiogram monitoring system. IEEE J. Trans. Eng. Health Med..

[49] Haahr R.G., Duun S., Thomsen E.V., Hoppe K., Branebjerg J. A wearable “electronic patch” for wireless continuous monitoring of chronically diseased patients. Proceedings of the International Summer School and Symposium on Medical Devices and Biosensors.

[50] Tsow F., Forzani E., Rai A., Wang R., Tsui R., Mastroianni S., Knobbe C., Gandolfi A.J., Tao N.J. (2009). A wearable and wireless sensor system for real-time monitoring of toxic environmental volatile organic compounds. IEEE Sens. J..

[51] López-Ruiz N., López-Torres J., Rodríguez M.Á.C., de Vargas-Sansalvador I.P., Martínez-Olmos A. (2015). Wearable system for monitoring of oxygen concentration in breath based on optical sensor. IEEE Sens. J..

[52] Pirbhulal S., Zhang H., Wu W., Mukhopadhyay S.C., Zhang Y. (2018). Heartbeats based biometric random binary sequences generation to secure wireless body sensor networks. IEEE Trans. Biomed. Eng..

[53] Granados J., Rahmani A.M., Nikander P. Towards energy-efficient HealthCare: An Internet-of-Things architecture using intelligent gateways. Proceedings of the International Conference on Wireless Mobile Communication and Healthcare—Transforming Healthcare Through Innovations in Mobile and Wireless Technologies.

[54] Rahmani A., Thanigaivelan N.K., Gia T.N. Smart e-Health Gateway: Bringing intelligence to Internet-of-Things based ubiquitous healthcare systems. Proceedings of the Annual IEEE Consumer Communications and Networking Conference.

[55] Crema C., Depari A., Flammini A. (2017). Virtual respiratory rate sensors: An example of a smartphone-based integrated and multiparametric mHealth gateway. IEEE Trans. Instrum. Meas..

[56] Kahn J.M., Barry J.R. (1997). Wireless infrared communications. Proc. IEEE.

[57] Han J., Zhang D., Cheng G., Guo L. (2015). Object detection in optical remote sensing images based on weakly supervised learning and high-level feature learning. IEEE Trans. Geosci. Remote Sens..

[58] Zhang D., Meng D., Han J. (2017). Co-saliency detection via a self-paced multiple-instance learning framework. IEEE Trans. Pattern Anal. Mach. Intell..

[59] Pahlavan K., Krishnamurthy P. (2002). Principles of Wireless Networks.

[60] Hasan M.K., Chowdhury M.Z., Shahjalal M., Jang Y.M. (2018). Fuzzy based network assignment and link-switching analysis in hybrid OCC/LiFi system. Wirel. Commun. Mob. Comput..

[61] Ross T.J. (2011). Fuzzy Logic with Engineering Applications.

[62] Kaya M., Alhajj R. (2006). Utilizing genetic algorithms to optimize membership functions for fuzzy weighted association rules mining. Appl. Intell..

[63] Acharya U.R. (2007). Heart rate variability. Advances in Cardiac Signal Processing.

[64] Zhang J. (2007). Effect of age and sex on heart rate variability in healthy subjects. J. Manip. Physiol. Ther..

[65] Chowdhury M.Z., Jang Y.M., Haas Z.J. (2011). Cost-effective frequency planning for capacity enhancement of femtocellular networks. Wirel. Pers. Commun..

